# AI-based detection of contrast-enhancing MRI lesions in patients with multiple sclerosis

**DOI:** 10.1186/s13244-023-01460-3

**Published:** 2023-07-16

**Authors:** Sarah Schlaeger, Suprosanna Shit, Paul Eichinger, Marco Hamann, Roland Opfer, Julia Krüger, Michael Dieckmeyer, Simon Schön, Mark Mühlau, Claus Zimmer, Jan S. Kirschke, Benedikt Wiestler, Dennis M. Hedderich

**Affiliations:** 1 Department of Diagnostic and Interventional Neuroradiology, School of Medicine, Klinikum rechts der Isar, Technical University of Munich, Ismaninger Str. 22, 81675 Munich, Germany; 2 jung diagnostics GmbH, Hamburg, Germany; 3 Department of Diagnostic, Interventional and Pediatric Radiology, Inselspital, University Hospital, University of Bern, Bern, Switzerland; 4 DIE RADIOLOGIE, Munich, Germany; 5 Department of Neurology, School of Medicine, Klinikum rechts der Isar, Technical University of Munich, Munich, Germany

**Keywords:** Magnetic resonance imaging, Multiple sclerosis, Contrast-enhancing lesions, Artificial intelligence, Clinical decision support

## Abstract

**Background:**

Contrast-enhancing (CE) lesions are an important finding on brain magnetic resonance imaging (MRI) in patients with multiple sclerosis (MS) but can be missed easily. Automated solutions for reliable CE lesion detection are emerging; however, independent validation of artificial intelligence (AI) tools in the clinical routine is still rare.

**Methods:**

A three-dimensional convolutional neural network for CE lesion segmentation was trained externally on 1488 datasets of 934 MS patients from 81 scanners using concatenated information from FLAIR and T1-weighted post-contrast imaging. This externally trained model was tested on an independent dataset comprising 504 T1-weighted post-contrast and FLAIR image datasets of MS patients from clinical routine. Two neuroradiologists (R1, R2) labeled CE lesions for gold standard definition in the clinical test dataset. The algorithmic output was evaluated on both patient- and lesion-level.

**Results:**

On a patient-level, recall, specificity, precision, and accuracy of the AI tool to predict patients with CE lesions were 0.75, 0.99, 0.91, and 0.96. The agreement between the AI tool and both readers was within the range of inter-rater agreement (Cohen’s kappa; AI vs. R1: 0.69; AI vs. R2: 0.76; R1 vs. R2: 0.76). On a lesion-level, false negative lesions were predominately found in infratentorial location, significantly smaller, and at lower contrast than true positive lesions (*p* < 0.05).

**Conclusions:**

AI-based identification of CE lesions on brain MRI is feasible, approaching human reader performance in independent clinical data and might be of help as a second reader in the neuroradiological assessment of active inflammation in MS patients.

**Critical relevance statement:**

Al-based detection of contrast-enhancing multiple sclerosis lesions approaches human reader performance, but careful visual inspection is still needed, especially for infratentorial, small and low-contrast lesions.

**Graphical Abstract:**

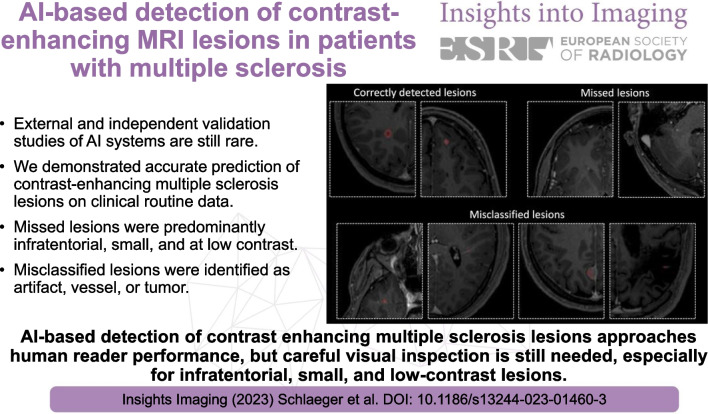

**Supplementary Information:**

The online version contains supplementary material available at 10.1186/s13244-023-01460-3.

## Key points

- Proper external validation studies of AI systems on unseen data from clinical routine are still rare.
- An externally developed AI tool accurately predicts contrast-enhancing lesions in clinical data of patients with multiple sclerosis.
- Missed lesions were predominately infratentorial, small, and at low contrast.
- False positive lesions represented alternative diagnoses such as artifacts, vessels, or tumor.
- AI-based detection of contrast-enhancing lesions is approaching human reader performance.

**Supplementary Information:**

The online version contains supplementary material available at 10.1186/s13244-023-01460-3.

## Background

Multiple sclerosis (MS) is the leading neuroinflammatory disease in the Western world and is associated with high morbidity, long-term disability and socioeconomic burden [1]. Magnetic resonance imaging (MRI) is a mainstay for the diagnostic work-up of patients with (suspected) MS [2]. By visualizing demyelinating and neurodegenerative processes in the central nervous system, MRI represents the key tool for diagnosis and monitoring of disease course and therapy in MS patients [2].

In the neuroradiological workup, major pathological changes in diseased nervous tissue of MS patients are focal areas of demyelination, so-called lesions [3]. Thereby, contrast-enhancing (CE) lesions point toward acute demyelinating processes, which has important implications for first-time diagnosis and changes in the therapeutic regime [4, 5]. Contrast enhancement characterizes lesions that are typically not older than eight weeks and is the key surrogate marker for active inflammation [4]. The detection of CE lesions next to non-enhancing lesions proves that brain white matter damage has occurred at multiple time points, referred to as dissemination in time, which is one fundamental diagnostic criterium for MS [6, 7]. Contrast enhancement is associated with the occurrence of clinical relapses [8] and the amount or volume of CE lesions is highly relevant for the evaluation of treatment efficacy [9]. Thus, an accurate and robust detection of CE lesions is critical for clinical decision making in MS patients.

Although CE lesions are an important finding on brain MRI of patients with MS, they may be easily missed by the radiologist. The morphology of CE lesions differs substantially between patients and scans with respect to size, shape (ring, punctual or linear enhancement), intensity and location [10]. Due to the steadily increasing patient throughput and the growing amount of imaging data, qualitative assessment of conventional MRI sequences comes along with non-negligible intra- and inter-observer variability having relevant implications for subsequent treatment decisions [11, 12]. Manual segmentation of CE lesions for further research or clinical questions remains a very time-consuming, tedious and error-prone task, which can become practically infeasible in clinical routine when dealing with large amounts of data under time constraints [11–13]. Consequently, fully automated detection and segmentation of CE lesions is highly desirable and might have an impact on the diagnostic workup of MS patients.

During the last decade, Artificial Intelligence (AI) and Machine Learning (ML) have made significant advances in medical imaging and have been lining up to enter the clinical workflow [14, 15]. Innovative AI techniques together with broader availability of digitalized data and advanced computer hardware promise to improve many routine radiological tasks such as acceleration of image acquisition, artifact reduction, and anomaly detection [16–21]. ML-based segmentation algorithms for identification and segmentation of brain lesions have significantly improved [12, 22–24]. Next to segmentation of white matter lesions on non-contrast MRI [25–28], AI systems allow to particularly detect CE lesions in MS brain tissue [10, 11, 29–31]. ML networks were applied to non-standardized and standardized (clinical trial) datasets for an automatic detection and delineation of CE lesions [10, 11, 29, 30] and deep learning (DL) networks can help to predict lesion enhancement based on non-contrast MRI [5].

High skepticism toward utility and applicability in the actual clinical setting remains, although such AI frameworks have been validated in their respective internal test setting, and generalizability and benchmarking were also assessed in large computational challenges such as the “WMH Segmentation Challenge 2017” (https://wmh.isi.uu.nl) [25]. Whether the published network performance is restricted to specific test environments and how the AI tool performs in real-world clinical scenarios are major questions that need to be addressed to overcome skepticism. Nevertheless, a proper external validation of AI systems in clinical routine is still rarely performed.

Therefore, in the present work, we aim to elucidate the potential of an externally developed and trained AI tool for CE lesion detection in our clinical setting.

We investigated the performance of an independently trained 3D convolutional neuronal network (CNN) for detection of CE lesions applied on brain images of MS patients in clinical routine, hypothesizing that the AI tool performs comparable to a human reader. With respect to two expert readers, we (i) assessed the potential of the automated AI tool for identifying patients with at least one CE lesion on a patient-level, and (ii) conducted a lesion-level analysis following a standardized reporting scheme to better understand the classifications made by the network.

## Methods

### Deep learning framework and external training

**Fig. 1:**
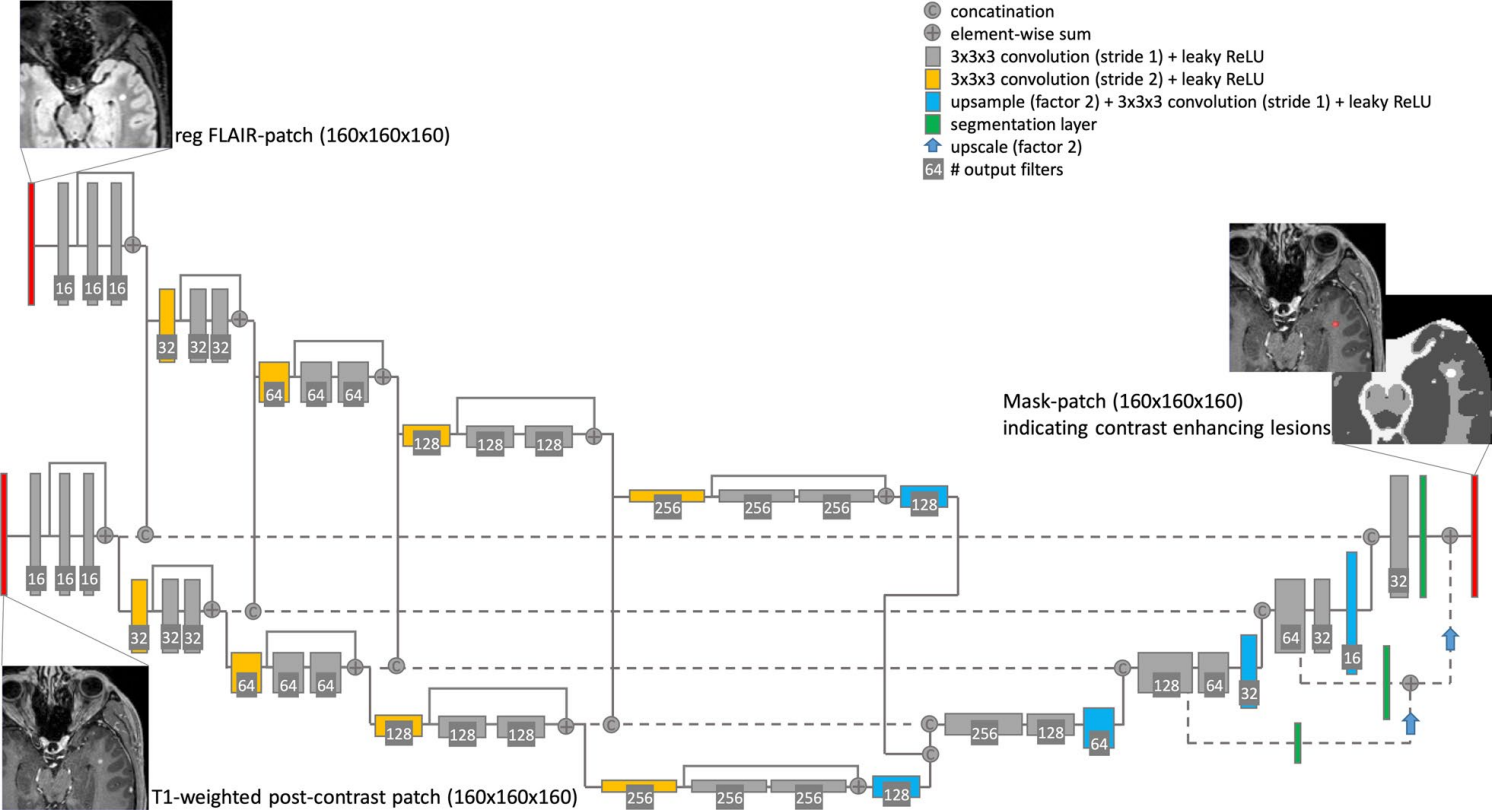
Architecture of the externally developed and trained 3D CNN with a fully convolutional encoder–decoder architecture with 3D convolutions, residual-block connections and four reductions of the feature map size. The two input images (T1-weighted post-contrast patch and registered FLAIR patch) were fed into the same encoder path with shared weights. Following every residual-block, the feature maps for the T1-weighted and the FLAIR input were concatenated and fed into the decoder. A segmentation mask was predicted, indicating contrast-enhancing lesions and background classes (grey matter, white matter, cerebrospinal fluid and FLAIR lesions). CNN, convolutional neural network; FLAIR, fluid-attenuated inversion recovery

A 3D CNN with a U-Net like encoder–decoder architecture (Fig. 1) was externally developed by jung diagnostics GmbH, Hamburg, Germany, and provided for external validation on our dataset.

A total of 1488 pairs of fluid-attenuated inversion recovery (FLAIR) and T1-weighted post-contrast image datasets of 934 MS patients originating from 81 different MRI scanners served as training data.

Two expert readers independently labelled CE lesions in the 1488 datasets (one trained radiology technologist with ten years of experience annotating MR images, and a PhD in neuroscience with ten years of experience in neuroimaging and lesion segmentation). A CE lesion was defined as a hyperintense area on the T1-weighted post-contrast images, on which enhancement could be punctual/filled or “ring-enhancing”. The enhancement must show a hyperintensity on the corresponding FLAIR. It was carefully verified that the hyperintensity is not caused by an artifact or by a normal anatomical structure that may cause a hyperintense signal (such as vessels).

For the DL framework, the heterogeneous input scans were re-sampled into an isometric 1 mm × 1 mm × 1 mm 3D-space and fed into the encoder in zero mean unit variance 160 × 160 × 160 patches. A rigid registration was used to register the corresponding FLAIR on the T1-weighted images beforehand. Information from FLAIR images was included in order to enhance network performance and reduce false positive rate. The two input patches were fed into the same encoder path with shared weights. Following every residual-block, the feature maps for the T1-weighted and the FLAIR input were concatenated and fed into the decoder. The encoder–decoder structure used was fully convolutional with 3 × 3 × 3 kernel size 3D convolutions. Four blocks with residual-block-connections reduced the spatial feature map four-times in the encoder path, before they got up-sampled to the original patch-size by the decoder. The output was six 3D probability masks at the same size as the original image, one for each segmentation that has been trained (CE lesions, FLAIR lesions, grey matter, white matter, cerebrospinal fluid, background). After the training, the final 3D CNN was transferred to the clinical institution which was responsible for the validation. Of note, the institution was not involved in the development and training phase and the training data did not include any scans from the MRI scanner used for testing.

### Clinical performance evaluation

The ability of the externally developed and trained AI tool to detect CE lesions was assessed on an independent test dataset of MS patients from clinical routine.

#### Study population

We retrospectively identified 359 MS patients (68.5% female; mean age 38.2 ± 10.3 years) with relapsing–remitting disease course (88%), clinically isolated syndrome (9.7%), primary progressive disease course (0.8%), secondary progressive disease course (0.8%), and radiologically isolated syndrome (0.8%) in our institutional PACS. Mean disease duration at baseline was 5.0 ± 4.4 years. This resulted in *n* = 504 datasets of baseline and follow-up scans acquired in our daily clinical practice consisting of FLAIR and T1-weighted post-contrast images originating from our clinical 3 T MRI scanner (Achieva, Philips Healthcare, Best, The Netherlands), respectively. The sequence parameters were implemented according to our side-specific clinical protocol (Table 1). The retrospective study was approved by the local institutional review board, and written informed consent was obtained from all patients.

**Table 1:** Sequence parameters

	FLAIR	T1-weighted post-contrast
Acquired voxel size [mm^3^]	1.03 × 1.03 × 1.5	1 × 1 × 1
Repetition time (TR) [ms]	10,000	9
Echo time (TE) [ms]	140	4
Acquisition time [min]	5	6
Acquisition plane	axial	sagittal

#### Gold standard definition via clinical reading

Two expert readers (one neuroradiologist with six years of experience: reader 1 (R1); and one neuroradiologist with eight years of experience: reader 2 (R2)) performed data annotation for gold standard definition. Each reader independently labelled CE lesions in a similar approach as performed for data annotation during external training in the 504 T1-weighted post-contrast sequences to estimate inter-observer variability. After independent labeling, all T1-weighted post-contrast images were reassessed by a consensus reading of both experts to define a ground truth (GT) CE lesion labeling.

#### Patient-level analysis

The AI tool created a binary segmentation mask indicating CE lesions for each dataset. The potential of the 3D CNN to identify patients with at least one CE lesion on a patient-level (CE (+) patient) in contrast to a CE(−) patient (no CE lesion) was compared to the clinical assessment by R1 and R2.

#### Lesion-level analysis

To further understand the classifications made by the 3D CNN, the AI segmentations of CE lesions were compared to the GT labeling of both readers. A sample of 50% of true positive (TP), false negative (FN) and false positive (FP) lesions was randomly selected and evaluated by one neuroradiologist with six years of experience. The following variables describing the CE lesions were evaluated: (specific) location, shape (ring, punctual or linear enhancement), maximum diameter and apparent contrast-to-noise ratio (aCNR). The aCNR [32] was calculated using the following equation:

1 $$a{\text{CNR}} = \frac{{\left( {{\text{SI}}_{{{\text{CE}}\,{\text{lesion}}}} - {\text{SI}}_{{{\text{NAWM}}}} } \right)}}{{{\text{SD}}\,{\text{of}}\,{\text{SI}}_{{{\text{NAWM}}}} }}$$

where SI_CE lesion_ is the signal intensity of the respective lesion, SI_NAWM_ is the signal intensity in a region of interest (ROI) placed in the surrounding normal-appearing white matter (NAWM), and SD of SI_NAWM_ is the corresponding standard deviation. In addition, a possible alternative diagnosis was noted for FP lesions.

### Statistical analysis

Statistical analysis was performed with SPSS (version 27.0, IBM SPSS Statistics for MacOS, IBM Corp.) and Python (Python Software Foundation, Python Language Reference, version 3.6, available at http://www.python.org). A *p* value of 0.05 was set as threshold for statistical significance.

On a patient-level, potential of the 3D CNN to correctly classify CE(+) patients was compared to the labeling by R1 and R2 using metrics for performance assessment: recall, specificity, precision, and accuracy. For comparison, inter-observer variability between R1 and R2 was assessed with recall, specificity, precision, and accuracy. To compare agreement between AI versus R1/R2 and inter-rater agreement, Cohen’s kappa values were calculated (AI vs. R1, AI vs. R2, and R1 vs. R2).

On a lesion-level, statistical significance of describing variables for TP and FN lesions was evaluated using the Pearson’s chi-squared test (for location and shape) and the Mann–Whitney *U* test (for diameter and aCNR).

## Results

The threshold to create a binary CE lesion segmentation mask based on the output 3D probability mask of the network was set to 0.98 in order to obtain an optimal trade-off between a relatively high lesion wise sensitivity at an acceptable false positive rate.

### Patient-level analysis

When compared to R1, the resulting recall for classification as CE(+) patient (at least one CE lesion) with the 3D CNN was 0.62 with a specificity of 0.99. 87% of classifications as CE(+) patient with the AI system were correct (precision). The accuracy was 0.94. Please refer to Additional file 1: Table SM1 for the corresponding confusion matrix.

When compared to R2, the resulting recall for classification as CE(+) patient (at least one CE lesion) with the 3D CNN was 0.75 with a specificity of 0.99. 91% of classifications as CE(+) patient with the AI system were correct (precision). The accuracy was 0.96. Please refer to Additional file 1: Table SM2 for the corresponding confusion matrix.

Thereby, particularly recall and specificity of AI versus R2 were comparable to R1 versus R2 (recall: 0.76; specificity: 0.98). Compared to R1, 88% of R2 classifications as CE(+) patient were correct (precision) with an accuracy of 0.95. Please refer to Additional file 1: Table SM3 for the corresponding confusion matrix.

In Table 2, all recall, specificity, precision, and accuracy values with 95% confidence intervals (CI) are shown.

**Table 2:** Results of the patient-level analysis

	Recall (95% CI)	Specificity (95% CI)	Precision (95% CI)	Accuracy
AI versus R1	0.62 (0.52–0.80)	0.99 (0.97–1.00)	0.87 (0.70–1.00)	0.94
AI versus R2	0.75 (0.55–0.88)	0.99 (0.97–1.00)	0.91 (0.76–0.99)	0.96
R1 versus R2	0.76 (0.63–0.92)	0.98 (0.97–1.00)	0.88 (0.82–0.98)	0.95

Recall, specificity, precision and accuracy of AI tool versus R1 and R2, as well as R1 versus R2

CI, confidence interval; AI, artificial intelligence; R1, reader 1; R2, reader 2

Cohen’s kappa for agreement between AI system and R1 was 0.69 (0.65–0.74) and between AI system and R2 was 0.76 (0.72–0.81)—both well within the range of agreement between R1 and R2 (Cohen’s kappa: 0.79 (0.75–0.83)).

According to the GT consensus reading of R1 and R2, of the CE(+) patients 46 patients only had one CE lesion. Of this patient cohort, the AI system detected 22 patients correctly and missed 24 patients.

### Lesion-level analysis

**Fig. 2:**
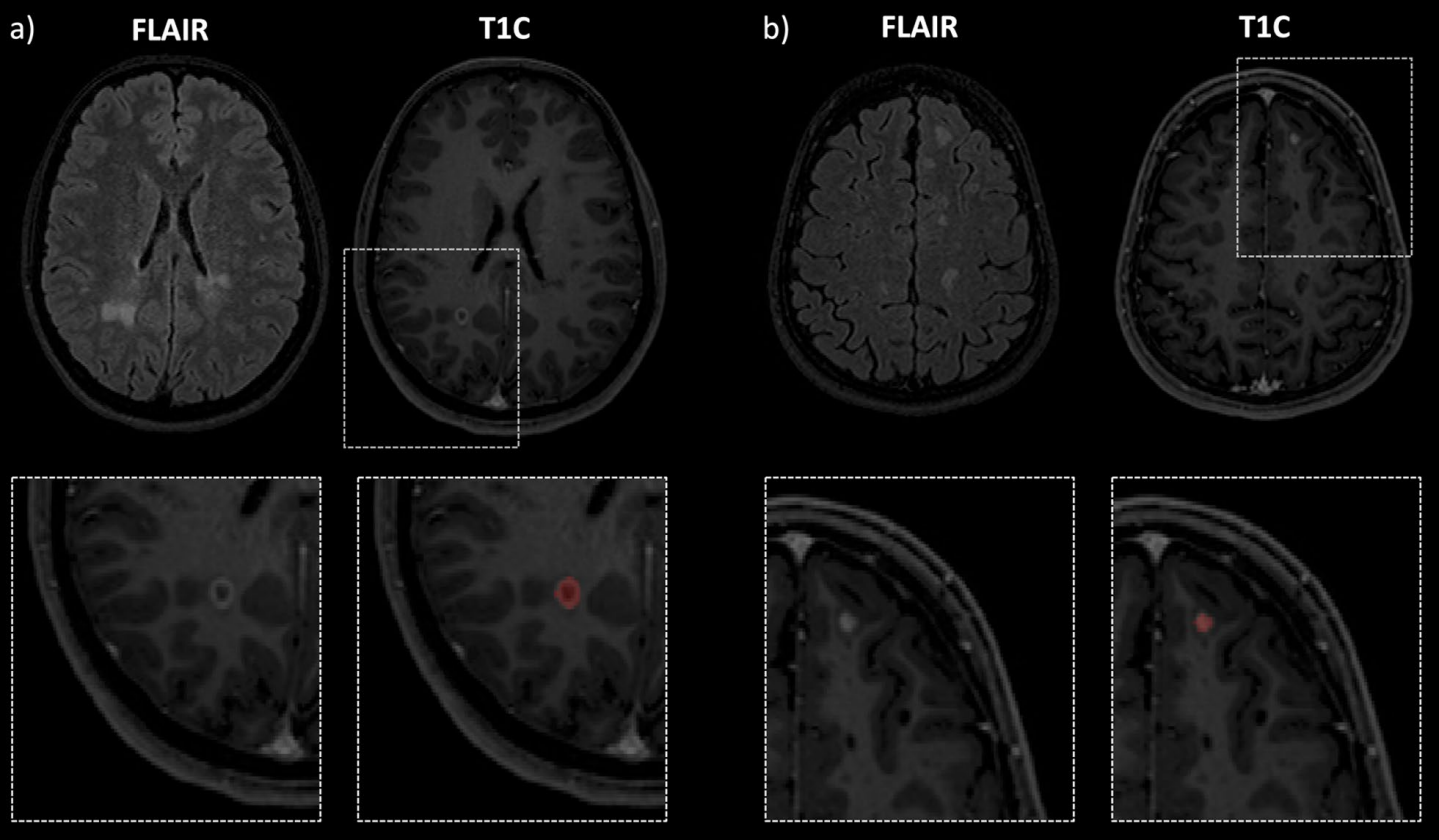
Two representative true positive lesions. The 3D CNN correctly labeled the subcortical, ring enhancing lesion in the right occipital lobe (**a**) and the juxtacortical, punctual enhancing lesion in the left frontal lobe (**b**). FLAIR and T1C as well as zoomed views of the CE lesions without and with segmentation masks are shown. CNN, convolutional neural network; T1C, T1-weighted post-contrast; CE, contrast-enhancing

**Fig. 3:**
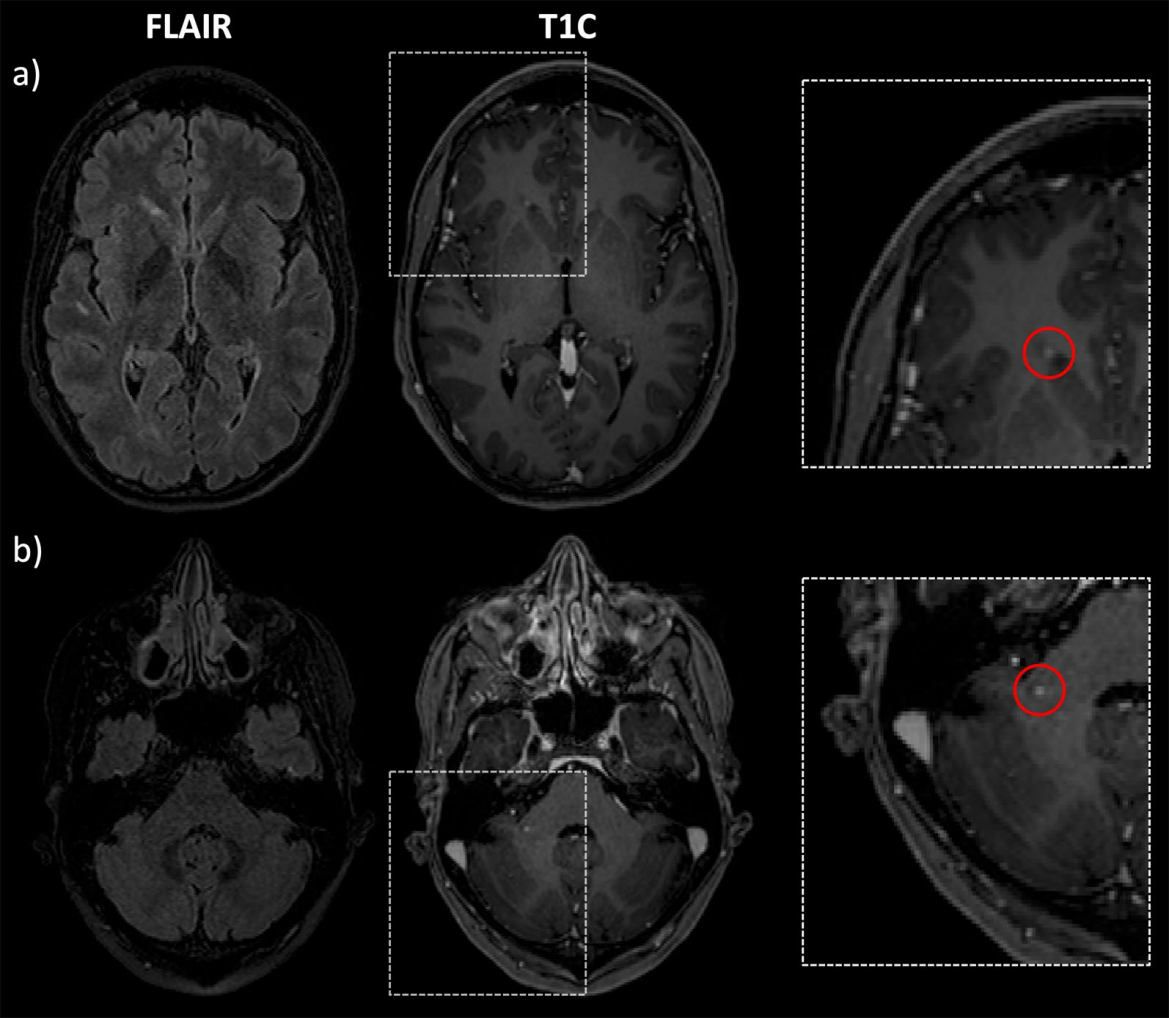
Two representative false negative lesions. Relatively small, low-contrast lesions (**a**) and predominantly lesions with infratentorial location (**b**) were missed by the 3D CNN. FLAIR and T1C as well as zoomed views of the CE lesions with annotation of the respective lesion are shown. CNN, convolutional neural network; T1C, T1-weighted post-contrast; CE, contrast-enhancing

**Fig. 4:**
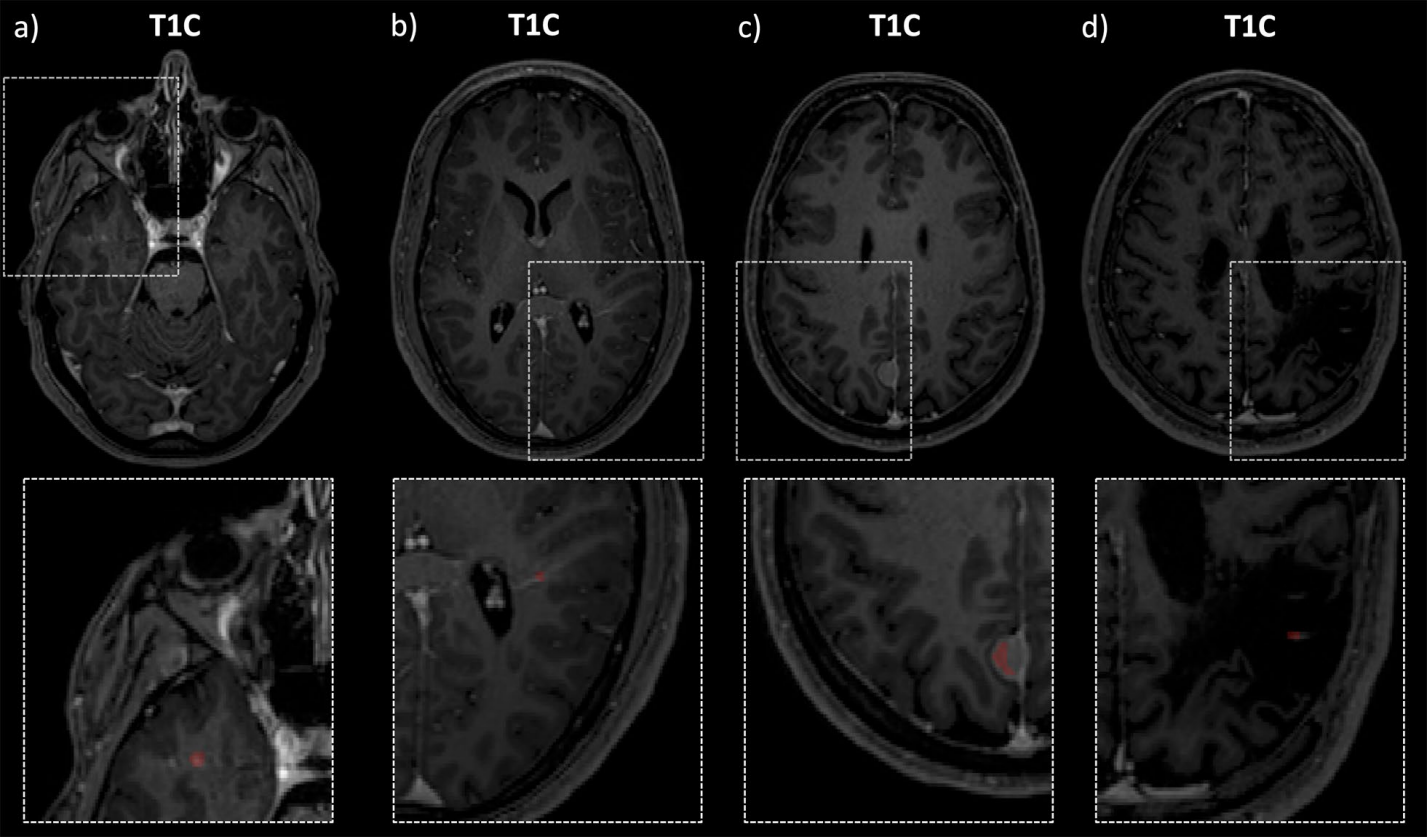
Four representative false positive lesions. Common alternative diagnoses were pulsation artifacts in the temporal lobe (**a**), vessel (**b**), meningioma (**c**), and hyperintensities in a tumor resection cavity (**d**). T1C as well as zoomed views of the CE lesions with segmentation masks are shown. T1C, T1-weighted post-contrast; CE, contrast-enhancing

The two expert readers labelled 164 CE lesions in total (GT labeling). When compared to the GT labeling on a lesion-level, the AI system detected 73 lesions correctly (TP), 91 lesions were missed (FN), and 22 lesions were misclassified as CE lesions (FP). Table 3 shows the results of the manual lesion-level analysis for TP and FN. FN lesions were predominantly located in the infratentorial location *(p* = 0.020). Relatively more TP lesions showed a ring enhancement, while FN lesions predominately showed punctual enhancement (*p* = 0.093). Mean aCNR was significantly lower in FN cases compared to TP cases (*p* = 0.001). Mean maximum diameter of FN lesions was significantly smaller compared to TP cases (*p* = 0.001). In Fig. 2, two representative TP lesion segmentations are shown. The 3D CNN correctly labeled the subcortical, ring enhancing lesion in the right occipital lobe (Fig. 2a) and the juxtacortical, punctual enhancing lesion in the left frontal lobe (Fig. 2b). Whereas in Fig. 3, typical FN lesions, that are relatively small, at low contrast, and often in the infratentorial location, are provided. Common alternative diagnoses for FP lesions were pulsation artifacts in the temporal lobe (*n* = 3), cerebral vessels (*n* = 2), meningioma (*n* = 2), and hyperintensities in a tumor resection cavity (*n* = 2) (Fig. 4).

**Table 3:** Results of the lesion-level analysis

	True positive CE lesions (*n* = 37)	False negative CE lesions (*n* = 46)	*p* value
Location			
(Juxta)Cortical	9	4	0.020*
Periventricular	10	13	
Infratentorial	1	11	
Subcortical	17	18	
Shape			
Ring enhancement	13	7	0.093
Punctual/filled	19	33	
Linear	5	6	
Diameter			
Mean; std	5.67; 2.09	3.52; 1.45	0.001*
aCNR			
Mean; std	20.37; 13.86	6.26; 17.02	0.001*

Variables describing true positive and false negative CE lesions are shown

CE, contrast-enhancing; std, standard deviation; aCNR, apparent contrast-to-noise ratio

*Indicates statistical significance (*p* < 0.05)

## Discussion

Our work demonstrates that the implementation of an externally developed and trained AI system for CE lesion detection can substantially contribute to the neuroradiological workup of MS patients in clinical routine. AI-based detection of CE lesions representing active inflammation in MS patients is feasible, approaching human reader performance with respect to recall, precision, and accuracy. Hence, lesions missed by the algorithm are rather small (< 4 mm) and at low contrast.

In current clinical practice, conventional MRI is a mainstay in the diagnostic workup of MS patients [2]. Disease activity is characterized by lesion load in the initial scan and the amount of newly formed lesions in follow-up scans [33]. Thereby, the detection of CE lesions is of outmost importance, as their presence demonstrates active inflammation and its suppression is the main target of current MS treatment [4, 5]. Consequently, in MRI examinations of MS patients, an accurate and reliable identification of CE lesions is crucial for an optimal patient care.

Particularly, for such complex tasks that require the precise analysis of large amounts of medical imaging data, AI frameworks promise to support the radiological reporting [34]. Recently, several AI tools for identification and delineation of CE MS lesions have been developed and presented [10, 11, 29–31]. However, their implementation and external validation in the real-world clinical setting is still rare.

In the present work, we translated an externally provided 3D CNN to our clinical routine. Our institution received the finalized AI framework without having been involved in the development and training phase, thus guaranteeing a truly independent test set. For testing, the algorithm was applied to routinely acquired MRI data from in-house MS patients. As publicly available clinical MRI datasets to test AI systems in the diagnostics of MS patients are scarce, studies often use datasets from large research studies for testing of their systems, which has important implications [5, 35]. First, the prevalence of the pathology of interest is often overrepresented. Second, most of the time, additional pathological changes on the MR images due to secondary diagnoses represent exclusion criteria. On the contrary, in our cohort, the percentage of patients with CE lesions was relatively low (15%) and thus, more realistic compared to values from other studies using clinical trial data (> 20%) [30, 36–39]. Moreover, the vast majority of FP lesions in our study represented alternative diagnoses such as meningiomas or tumor resection cavities, which would be excluded in large research studies, although reflecting the true clinical patient population. Consequently, we could investigate the applicability of an externally provided AI tool in our clinical routine by testing in a real-word clinical scenario rather than under artificial “laboratory” conditions.

Our findings highlight that AI-based identification of MS patients with CE lesions and thus, active inflammation is comparable to human reader performance. The agreement between the AI segmentation and human reader labeling of CE lesions was in the range of inter-observer variability of the two expert readers. Among the unidentified CE(+) patients were mainly patients with only a single CE lesion. In the clinical setting, the AI system might replace the typically performed second reading by another radiologist and consequently significantly speed up the neuroradiological workflow. Of note, the agreement between the AI system and R2 is better than between the AI system and R1, which might be due to the slightly greater clinical experience of R2. The remaining inter-observer variability between R1 and R2 underlines how difficult and subjective an accurate manual annotation of CE lesions is, which on the one hand means a challenge in developing algorithms with high accuracy, however, on the other hand, underlines the need for robust AI tools to objectively perform this task [10]. On a patient-level, we concentrated our analysis on the performance of the binary classification as CE(+) patient (with at least one CE lesion) and CE(−) patient (no CE lesion), as assessment of active inflammation (represented by at least one CE lesion) is one of the main tasks for radiologists in MS imaging.

The applied 3D CNN incorporates information from T1-weighted post-contrast and FLAIR sequences. Considering that CE lesions also appear hyperintense on FLAIR images [11, 40] and thus, excluding non-lesion enhancement (e.g., in vessels) might be the cause for the extremely low number of FP classifications as CE(+) patient (around 1% compared to both readers) despite the clinically very heterogeneous patient collective.

Whereas the 3D CNN is reliably identifying ring enhancing and bigger (> 5.5 mm) lesions, the vast majority of missed lesions was rather small and at low contrast. Other AI frameworks for CE lesion identification report similar problems with the detection of small lesions [10, 11, 30]. Particularly small lesions mean a challenge also for human readers, which is reflected by high inter-observer variability, and affects accurate labeling of training datasets. Incorporating multiple radiologists in the labeling task for training data might help to overcome this challenge [10]. Additionally, in our clinical cohort, the AI system predominantly missed infratentorial lesions. The identification of infratentorial CE lesion is particularly challenging [41]. The posterior cranial fossa is known to be prone to artifacts due to the surrounding structures filled with air or consisting of bone, which compromises CE lesion detection in this area in general. Additionally, in adults, infratentorial lesions are less common than supratentorial lesions [42], which might lead to an underrepresentation of these lesions in the training dataset, consequently impacting the overall network performance.

As MRI has been revolutionizing medical imaging and patient care for at least four decades now, with ever faster, more robust, and specialized acquisition techniques, the patient load and amount of available imaging data is exponentially growing. To still be able to handle the provided big data, radiologists can rely on support of an increasing pool of AI algorithms which promise to help during the image reporting process [24]. In order to bridge the gap between development of these AI frameworks in controlled research settings and their implementation in real-world clinical practice, studies that validate the algorithms in daily routine are indispensable. Our work contributes to the translation of high-quality AI tools to the actual radiological workup. Future studies relating clinical outcomes to the network performance are necessary.

The present work is not without limitations. First, the two expert readers had different clinical experience, which might account for some inter-observer variability. Second, the overall accuracy might be affected by class imbalance because of the considerably low number of patients with CE lesions (15%). Third, on a patient-level, datasets are not completely independent as multiple follow-up scans of some patients are included in the dataset. However, the dataset reflects an actual clinical patient population with baseline and follow-up scans. Fourth, also on the lesion-level, individual lesions cannot be treated as though they were statistically independent as several patients contributed multiple lesions to the analysis. Consequently, lesions from the same patient might appear more uniform with respect to the evaluated lesion describing variables (location, shape, diameter, and aCNR); however, this was not subject of the present work.

## Conclusion

In conclusion, we could confirm that the implementation of an externally developed and trained AI tool for CE lesion detection in MS patients in our clinical routine is feasible and valuable, approaching human reader performance with respect to recall, precision, and accuracy. In the future, the AI tool might be a potential alternative to a second reader in the neuroradiological assessment of active inflammation in MS patients.

## Supplementary Information

**Additional file 1.** Table SM1, Table SM2, and Table SM3 provide confusion matrices for AI versus R1/R2 and R1 versus R2 for the classification as CE(+) patient or CE(-) patient (patient-level analysis). Table SM4 provides results of an additional lesion-level analysis concerning lobe location of supratentorial CE lesions.

## Data Availability

The datasets used and/or analyzed during the current study are available from the corresponding author on reasonable request.

## Abbreviations

aCNR: Apparent contrast-to-noise ratio
AI: Artificial Intelligence
CE: Contrast-enhancing
CNN: Convolutional neuronal network
DL: Deep learning
FLAIR: Fluid-attenuated inversion recovery
FN: False negative
FP: False positive
GT: Ground truth
ML: Machine Learning
MRI: Magnetic resonance imaging
MS: Multiple sclerosis
R1: Reader 1
R2: Reader 2
std: Standard deviation
TP: True positive
