# Supplementary material for: AI-based detection of contrast-enhancing MRI lesions in patients with multiple sclerosis
Source: Insights Imaging. 2023 Jul 16;14:123. doi: 10.1186/s13244-023-01460-3 (PMC10350445; doi:10.1186/s13244-023-01460-3)
Supplement: Supplementary file 1 — Additional file 1. Table SM1, Table SM2, and Table SM3 provide confusion matrices for AI versus R1/R2 and R1 versus R2 for the classification as CE(+) patient or CE(-) patient (patient-level analysis). Table SM4 provides results of an additional lesion-level analysis concerning lobe location of supratentorial CE lesions. [file 13244_2023_1460_MOESM1_ESM.pdf]

AI-based detection of contrast-enhancing MRI lesions in patients with multiple sclerosis

ELECTRONIC SUPPLEMENTARY MATERIAL

Table SM1: Results of the patient-level analysis. Confusion matrix reader 1 versus AI tool for the classification as CE(+) patient or CE(-) patient.

	**Reader 1: CE(+) patient**	**Reader 1: CE(-) patient**	
**AI: CE(+) patient**	41	6	47
**AI: CE(-) patient**	25	432	457
	66	438	504

AI, artificial intelligence; CE, contrast-enhancing; CE(+), patient with at least one CE lesion; CE(-), patient with no CE lesion.

Table SM2: Results of the patient-level analysis. Confusion matrix reader 2 versus AI tool for the classification as CE(+) patient or CE(-) patient.

	**Reader 2: CE(+) patient**	**Reader 2: CE(-) patient**	
**AI: CE(+) patient**	43	4	47
**AI: CE(-) patient**	14	443	457
	57	447	504

AI, artificial intelligence; CE, contrast-enhancing; CE(+), patient with at least one CE lesion; CE(-), patient with no CE lesion.

Table SM3: Results of the patient-level analysis. Confusion matrix reader 1 versus reader 2 for the classification as CE(+) patient or CE(-) patient.

	Reader 1: CE(+) patient	Reader 1: CE(-) patient	
**Reader 2: CE(+) patient**	50	7	57
**Reader 2: CE(-) patient**	16	431	447
	66	438	504

CE, contrast-enhancing; CE(+), patient with at least one CE lesion; CE(-), patient with no CE lesion.

Table SM4: Additional results of the lesion-level analysis. Only supratentorial lesions from Table 3 in the manuscript were included. The lobe location was not significantly different between true positive and false negative CE lesions. Statistical significance was evaluated using the Pearson’s chi-squared test, with $p < 0.05$ as threshold for statistical significance.

		True positive CE lesions (n = 36)	False negative CE lesions (n = 35)	***p*-value**
**Lobe**	**Frontal**	20	20	0.596
	**Parietal**	9	5	
	**Temporal**	2	4	
	**Occipital**	5	6	

CE, contrast-enhancing.
